# Hypoxic Preconditioning Improves Survival of Cardiac Progenitor Cells: Role of Stromal Cell Derived Factor-1α–CXCR4 Axis

**DOI:** 10.1371/journal.pone.0037948

**Published:** 2012-07-18

**Authors:** Fengdi Yan, Yuyu Yao, Lijuan Chen, Yefei Li, Zulong Sheng, Genshan Ma

**Affiliations:** Department of Cardiology, Zhongda Hospital, Medical School of Southeast University, Nanjing, Jiangsu, China; Northwestern University, United States of America

## Abstract

**Background:**

Cardiac progenitor cells (CPCs) have been shown to be suitable in stem cell therapy for resurrecting damaged myocardium, but poor retention of transplanted cells in the ischemic myocardium causes ineffective cell therapy. Hypoxic preconditioning of cells can increase the expression of CXCR4 and pro-survival genes to promote better cell survival; however, it is unknown whether hypoxia preconditioning will influence the survival and retention of CPCs via the SDF-1α/CXCR4 axis.

**Methods and Results:**

CPCs were isolated from adult mouse hearts and purified by magnetic activated cell sorting using c-kit magnetic beads. These cells were cultured at various times in either normoxic or hypoxic conditions, and cell survival was analyzed using flow cytometry and the expression of hypoxia-inducible factor-1α (HIF-1α), CXCR4, phosphorylated Akt and Bcl-2 were measured by Western blot. Results showed that the expression of pro-survival genes significantly increased after hypoxia treatment, especially in cells cultured in hypoxic conditions for six hours. Upon completion of hypoxia preconditioning from c-kit+ CPCs for six hours, the anti-apoptosis, migration and cardiac repair potential were evaluated. Results showed a significant enhancement in anti-apoptosis and migration *in vitro*, and better survival and cardiac function after being transplanted into acute myocardial infarction (MI) mice *in vivo*. The beneficial effects induced by hypoxia preconditioning of c-kit+ CPCs could largely be blocked by the addition of CXCR4 selective antagonist AMD3100.

**Conclusions:**

Hypoxic preconditioning may improve the survival and retention of c-kit+ CPCs in the ischemic heart tissue through activating the SDF-1α/CXCR4 axis and the downstream anti-apoptosis pathway. Strategies targeting this aspect may enhance the effectiveness of cell-based cardiac regenerative therapy.

## Introduction

Recent studies have accumulated compelling evidence suggesting not only that the adult heart has regeneration potential [1], but even the aging [2] and diseased heart [3] shows regenerative success. The heart should no longer be regarded as a terminally differentiated organ. In mice, but more importantly in human beings, there are several populations of cardiac progenitor cells (CPCs) that reside in the heart [3]–[7]. These resident CPCs are self-renewing, clonogenic, multipotent and have the ability to proliferate and spontaneously differentiate into either functional cardiomyocytes, smooth muscle cells or endothelial cells *in vitro* and *in vivo* [4]–[7]. Among these CPCs, c-kit positive cardiac progenitor cells have shown to be suitable in cell therapy for resurrecting injured myocardium [4], [5]. Ischemic heart disease is one of the leading causes of morbidity and mortality in the industrialized world [8]. Recently, stem cell therapy has emerged as a potent new strategy for regenerating damaged myocardium. Previous studies have already shown that transplantation of stem/progenitor cells can improve cardiac function [4], [5], [9], [10]. Among those cells, cardiac stem/progenitor cells have gained the most interest because they are an endogenous component of the heart and directly regenerate myocardium and blood vessels [9]–[11]. Cardiac stem/progenitor cells also present beneficial autocrine and paracrine effects after their engraftment [12], [13].

Despite the significant advances in cell therapy the poor retention of transplanted stem/progenitor cells poses a major barrier to the effectiveness of stem cell therapy [14], [15]. Strategies aimed to enhance the survival of transplanted cells have attracted significant attention. The survival and proliferation of transplanted progenitor cells in ischemic heart tissue would require cell adaptation to the harsh, low oxygen tension environment [16]. Previous reports have demonstrated that hypoxia preconditioning can increase the ability of transplanted stem/progenitor cells to survive and proliferate ability of *in vitro* and their therapeutic effects *in vivo* [17]–[19]. However, the mechanisms underlying the beneficial effects of hypoxia preconditioned progenitor cells remain incomplete.

Accumulating evidence has indicated that stromal cell-derived factor-1α (SDF-1α) is increased in the ischemic heart and initiates cardioprotective effects. The pretreatment of bone marrow derived mesenchymal stem/progenitor cells with SDF-1α enhances engraftment and improves cardiac function after myocardial infarction [20], [21]. SDF-1α produces these effects as the unique ligand for its receptor, CXCR4 [22], [23], and SDF-1α/CXCR4 axis signaling can activate Akt and the promotion of cell survival and proliferation [20], [24]–[26]. Previous studies have shown that hypoxia preconditioning can up-regulate the expression of CXCR4 in cardiac progenitor cells [10]. However, it is not clear whether hypoxia preconditioning improves the survival of cardiac progenitor cells, and whether the protective effects are operated via the SDF-1/CXCR4 axis.

The present study demonstrated that hypoxic preconditioning markedly improved cardiac progenitor cell survival and enhanced the beneficial effects of cell therapy for repairing the damaged heart.

## Results

### CPCs Generation and Phenotypic Characterization

**Figure 1:**
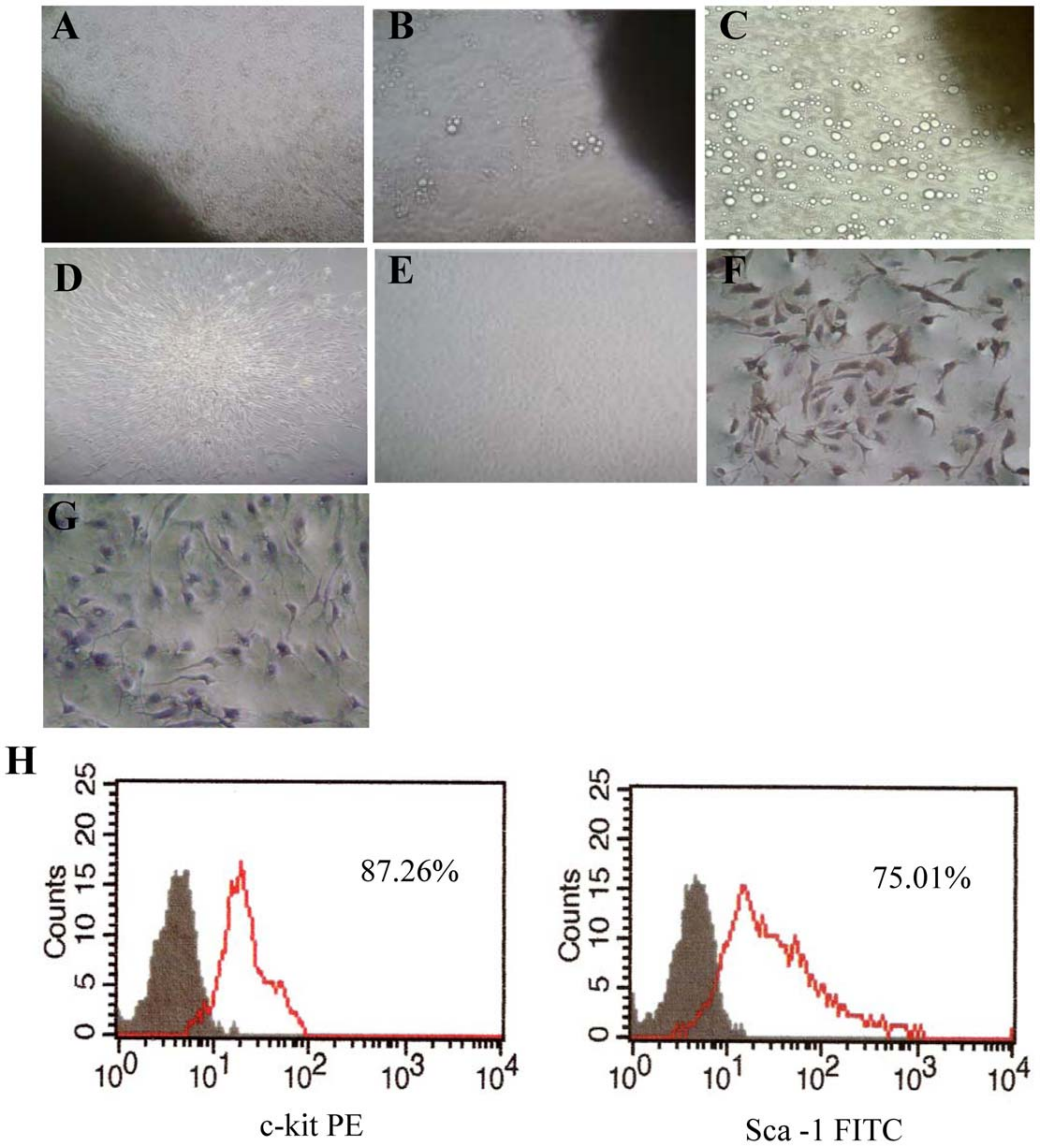
Characterization of cultured CPCs. (A) Typical phase-contrast images of the outgrowth from enzymatically digested cardiac explants after planted for 1 h (×100 magnification). (B) Cells (small, round, phase-bright) migrated from the cardiac explants, aggregated and proliferated over the fibroblast layer after culturing for ten days (×100 magnification). (C) Cells migrated from the cardiac explants after culturing for 14 days (×200 magnification). (D) Representative clone generated by CPCs (×200 magnification). (E) Phase-contrast image of CPCs after passage (×100 magnification). (F) Representative immunocytochemistry staining of CPCs for expression of cell surface marker c-kit. (×200 magnification) (G) Negative control immunocytochemistry staining of c-kit. (H) Representative flow cytometric analyses of CPCs for expression of the cell surface markers c-kit, Sca-1.

CPCs were obtained with mild enzymatic digestion from adult C57BL/6 mouse hearts, and enriched for c-kit+ cells by using magnetic-activated cell sorting. After ten days in culture, a layer of fibroblast-like cells emerged from adherent plated adult mouse heart tissues (named cardiac explants) and small, round and phase-bright cells migrated (Figure 1B and 1C). Inverted phase contrast microscope examinations showed that CPCs presented clone-like proliferation (Figure 1D). The obtained CPCs could differentiate spontaneously into pulsing cardiomyocytes (Movie S1). Cell surface marker expression was analyzed by immunocytochemistry staining of c-kit (Figure 1F). The purity of sorted c-kit+ CPCs was characterized by flow cytometric analyses of the cell surface marker c-kit and Sca-1(Figure 1G). Before magnetic separation, 6.5% of cardiosphere derived cells were positive for c-kit expression, while more than 85% were positive for c-kit expression after cell sorting (Figure 1G).

### Effects of Hypoxia Preconditioning on CPCs

**Figure 2:**
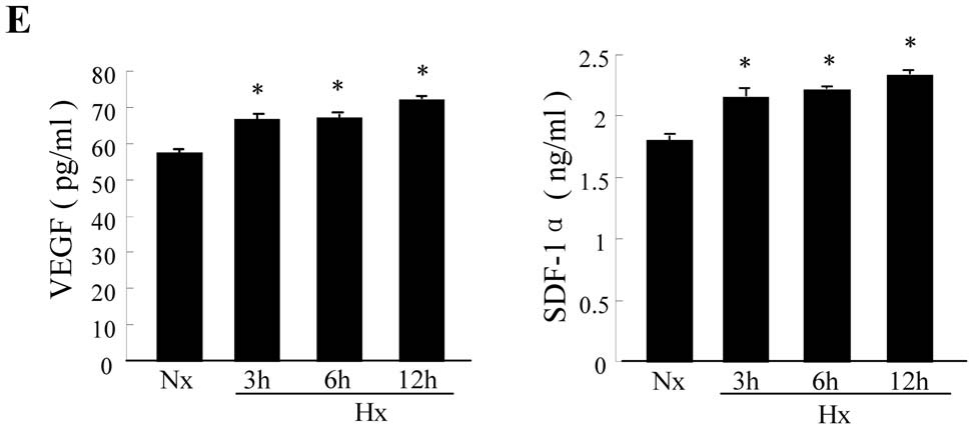
Effects of hypoxia preconditioning on CPCs *in vitro*. CPCs were cultured under either normoxia (Nx) or hypoxia (Hx) for 3, 6,12, and 24 hours. (A) Flow cytometry analysis of apoptosis in CPCs cultured under either normoxia or hypoxia at different times. (B) Quantitative analysis of apoptotic cells identified by flow cytometry. Data were obtained from three independent experiments and are expressed as mean ± SD. *P<0.01 vs. normoxia (Nx) group. (C) Representative Western blots of HIF-1α, CXCR4, and β-actin protein expression in CPCs (top); quantification of HIF-1α and CXCR4 protein levers (normalized to β-actin protein levels ). (D) Representative Western blots of p-Akt, Bcl-2 and β-actin protein expression in CPCs (top); quantification of p-Akt and Bcl-2 protein levers (normalized to β-actin protein levels). (E) Supernatant VEGF and SDF-1α concentrations measured by ELISA in CPCs that underwent hypoxia preconditioning. Values are mean ± SD. n = 3. *P<0.01vs.normoxia.

The viability of c-kit+ CPCs was measured by flow cytometry after being cultured under normoxic and hypoxic conditions for 3 h, 6 h, 12 h, and 24 h. There were no significant differences in the apoptosis rate between the normoxia and hypoxia 3 h, 6 h, and 12 h groups, especially in the hypoxia 6 h group (normoxia: 4.67±0.45; Hypoxia 6 h: 4.47±0.31). The rate of apoptosis increased when incubated in hypoxic conditions for 24 h (normoxia: 4.67±0.45; hypoxia 24 h: 7.43±0.61; *P*<0.01) (Figure 2A, B). There was also a trend of apoptosis in c-kit+ CPCs when cultured in hypoxic conditions for more than 12 h.

We further assessed hypoxia associated protein expression via Western blot and found that the expression of HIF-1α and CXCR4 was increased and presented a time-dependent manner (Figure 2D). Since Akt and Bcl-2 play a critical role in inhibiting apoptosis and improving cell survival, we examined the protein expression of phosphorylated Akt and Bcl-2. The results presented an obvious increase in hypoxia preconditioned c-kit+ CPCs. Compared with normoxia accompanied with the elevation of p-Akt protein expression (Figure 2E), the Bcl-2 protein expression in CPCs was elevated three fold after hypoxia treatment for six hours.(normoxia: 0.347±0.012; hypoxia 6 h: 0.968±0.005; *P*<0.01). Hypoxia preconditioning of c-kit+ CPCs could enhance their anti-apoptosis ability and improve their survival.

Since hypoxia preconditioning may influence the potential autocrine and paracrine mechanisms induced by c-kit+ CPCs, we conducted an ELISA to measure the levels of VEGF and SDF-1α released from c-kit+ CPCs. The results showed that VEGF and SDF-1α significantly increased after hypoxic preconditioning compared with normoxia-cultured c-kit+ CPCs (Figure 2F). These data indicate that hypoxic preconditioning for 6 h would be the optimal time course for preparing c-kit+ CPCs in our experiments.

### CPCs Migration and Anti-apoptosis

**Figure 3:**
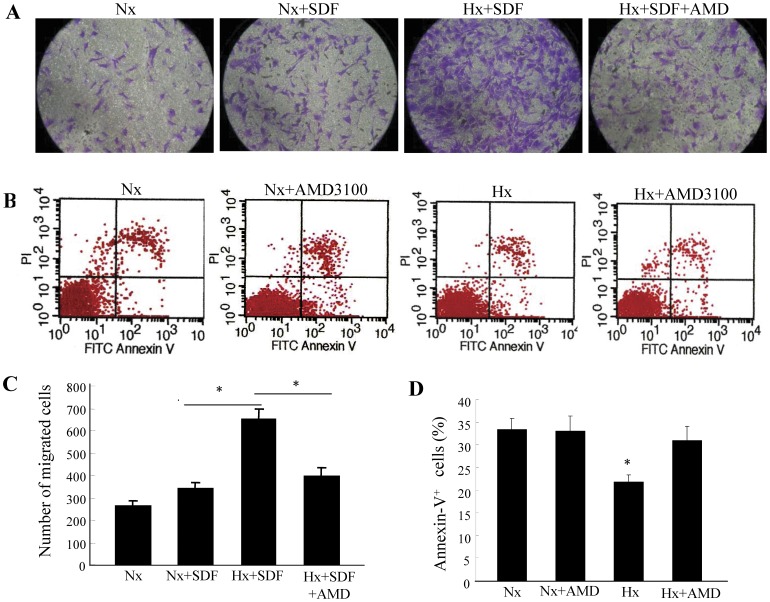
CPC migration and survival influenced by hypoxia preconditioning. CPCs were cultured under either normoxic (Nx) or hypoxic (Hx) conditions for six hours with or without AMD3100 (CXCR4-selective antagonist, 5 µg/mL). (A) Representative migrated CPCs (stained with crystal violet) are shown (×200 magnification). (C) Quantitative analysis of migrated cells. Data are presented as mean ± SD. n = 5. *P<0.01. (B) CPCs were exposed to hypoxia along with serum deprivation for 48 hours. Representative flow cytometry analysis of apoptotic cells after being labeled with annexin V and propidium iodide. (D) Quantitative analysis of apoptosis evaluated by annexin V staining. Data were obtained from five independent experiments and are expressed as mean ± SD. *P<0.01 vs. normoxia (Nx) group & normoxia (Nx) + AMD group & hypoxia (Hx) + AMD group.

To determine whether hypoxia preconditioning will influence CPC mobility toward SDF-1, we performed an *in vitro* migration assay, and the c-kit+ CPCs were placed under either normoxic or hypoxic conditions with or without the CXCR4 specific antagonist AMD3100 for 6 h prior to the migration assay. Hypoxic preconditioning of c-kit+ CPCs significantly increased migration toward SDF-1, but this effect was abolished when hypoxia preconditioned CPCs were incubated with AMD3100 (Figure 3A, C).

The effect of hypoxia preconditioning on cell anti-apoptosis was assessed by flow cytometry analysis after labeled with annexin V and propidium iodide. Cell apoptosis was induced via prolonged hypoxia for 48 hours along with serum deprivation. It showed a reduction in apoptosis on c-kit+ CPCs that were hypoxia preconditioned for six hours compared with those cultured in normoxic conditions. The protective effects of hypoxia preconditioning were largely blocked when preconditioned c-kit+ CPCs were incubated with the CXCR4-selective antagonist AMD3100 (Figure 3 B,D).

### Effect of Hypoxia Preconditioned CPCs in Healing MI

**Figure 4:**
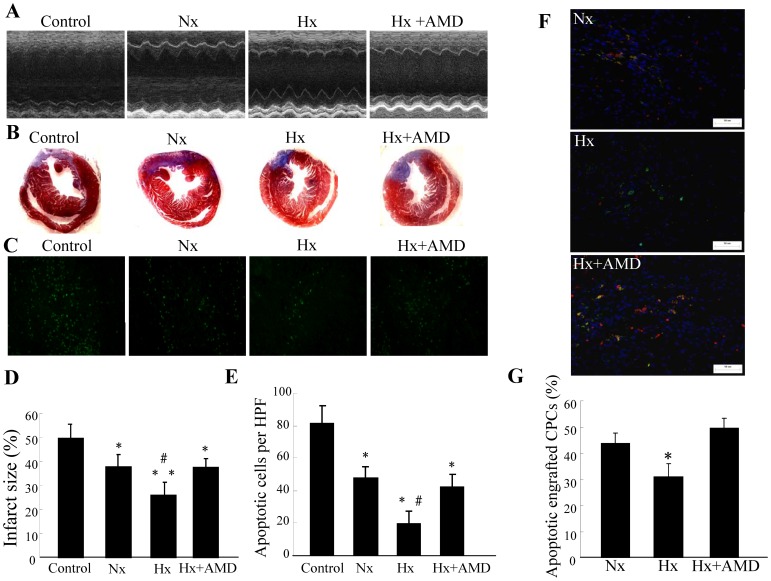
Effect of hypoxia preconditioned CPCs in treating MI. CPCs were cultured under either normoxia (Nx) or hypoxia (Hx) for six hours with or without AMD3100 (CXCR4-selective antagonist, 5 µg/mL) and then intramyocardially injected into mice after surgical MI. Mice in the control group were administered DMEM. (A) Echocardiographic measurements were performed seven days after CPC transplantation. Representative M-mode images at the level of papillary muscles were recorded. (B) Representative Masson’s trichrome staining of transverse heart sections seven days after coronary ligation and CPC administration. (C) Representative phase micrographs show TUNEL-positive apoptotic cells in bordering myocardium adjacent to the infarct zone seven days after coronary ligation (×200 magnification). (D) Quantitative analysis of infarct size. Values are expressed as mean ± SD. n = 5. *P<0.05 vs. control group, **P<0.01 vs. control group, # *P*<0.05 vs. normoxia (Nx) & hypoxia (Hx) + AMD group. (E) Quantitative analysis of apoptotic cells in bordering myocardium. Values are expressed as mean ± SD. n = 5. *P<0.01 vs. control group, ^#^ *P*<0.01 vs. normoxia (Nx) & hypoxia (Hx) + AMD group. (F) Representative TUNEL+ apoptotic engrafted CPCs in heart tissues which were collected 72 h after cell transplantation (×200 magnification). (G) The percentage of apoptotic engrafted CPCs. The number of apoptotic engrafted CPCs was assessed by double positive of GFP+/TUNEL+ (yellow). Values are expressed as mean ± SD. n = 5. *P<0.01 vs. normoxia (Nx) group & hypoxia (Hx) + AMD group.

After observing the beneficial effects induced by hypoxia preconditioning of c-kit+ CPCs *in vitro*, we further investigated preconditioning *in vivo*. We administered c-kit+ CPCs via direct intramyocardial injection shortly after surgical MI, and cardiac functions were measured seven days later by transthoracic echocardiography. Hearts were later used for histological analyses. Echocardiographic results showed that the LVEF and LVFS were both significantly greater in hearts administrated with c-kit+ CPCs. Mice transplanted with hypoxia preconditioned c-kit+ CPCs demonstrated greater cardiac function improvement than those treated with normoxic cultured CPCs. The enhancement was abolished when hypoxia preconditioned c-kit+ CPCs were incubated with CXCR4 antagonist AMD3100 (Figure 4 A, Table 1).

**Table 1:** Left ventricular function measured by echocardiography and infarct size among groups 7 days after CPCs transplantation.

	Control	Nx	Hx	Hx+AMD
LVEF (%)	30.5±3.7	44.7±3.1*	54.9±4.5*	43.7±3.7*
LVFS (%)	14.3±2.0	21.9±1.6*	27.9±2.8*	21.3±1.8*
Infarct size (%)	49.6±5.9	37.9±5.0*	25.8±5.4* §	37.5±3.7*
BW(g)	23.3±0.9	23.3±0.4	24.8±0.6#	23.6±0.9
HW(mg)	132.6±11.1	127.4±6.6	117.0±10.4	127.2±7.4
HW/BW(mg/g)	5.7±0.4	5.5±0.3	4.7±0.4* §	5.4±0.5

Date represented as mean ± SD; n = 5 in each group.

*Nx* normoxia, *Hx* hypoxia, *AMD* AMD3100, *LVEF* left ventricular ejection fraction, *LVFS* left ventricular fractional shortening, *BW* body weight, *HW* heart weight, *HW/BW* heart weight/body weight.

* *P*<0.01 vs. Control

§ *P*<0.05 vs. Nx and Hx + AMD

# *P*<0.05 vs. Control and Nx.

There were no apparent differences in heart weights among the four groups, whereas the heart weight/body weight ratio decreased in mice receiving hypoxia preconditioned c-kit+ CPCs compared with every other group (Table 1). Infarct size was analyzed via Masson trichrome staining and there was a reduction in infarct size in hearts from mice administered with hypoxia preconditioned c-kit+ CPCs (Figure 4 B, D).

TUNEL-positive nuclei were lower in the peri-infarct myocardium in hearts from mice administered hypoxia preconditioned c-kit+ CPCs than in mice treated with normoxia-cultured c-kit+ CPCs and the control group (hypoxia: 19.6±7.5; normoxia: 47.8±6.5; control 81.2±11.3; *P*<0.01) (Figure 4 C, E); also, these apparent beneficial effects induced by hypoxia preconditioning were mostly blocked when preconditioned CPCs were incubated with AMD3100(hypoxia: 19.6±7.5; hypoxia + AMD3100: 42.4±7.7; *P* = 0.004). Moreover, hypoxia preconditioned CPCs had shown better survival than CPCs cultured in normoxia after transplanted into the damaged myocardium. Apoptotic transplanted CPCs was evaluated by double positive of GFP+/TUNEL+ (yellow) staining. Our results demonstrated that hypoxia preconditioned CPCs survived better than CPCs cultured in normoxia (hypoxia: 30.9±5.1; normoxia: 43.8±4.0; *P* = 0.002); also, these obvious beneficial effects induced by hypoxia preconditioning were mostly abolished when preconditioned CPCs were incubated with AMD3100 (hypoxia: 30.9±5.1; hypoxia + AMD3100: 49.7±3.7; *P*<0.001) (Figure 4 F, G), and there is no significant difference between the normoxia group and hypoxia+AMD3100 group (*P* = 0.161). These results indicate that the therapeutic effects of c-kit+ CPC transplantation in a healing MI were enhanced by hypoxia preconditioning via SDF-1/CXCR4 axis.

## Discussion

The key findings in our study are listed as the following: (1) An adequate amount of c-kit positive CPCs could be obtained from adult mouse heart tissue using enzymatic digestion and purified with magnetic-activated cell sorting; (2) Hypoxia preconditioning of c-kit+ CPCs could not only significantly augment anti-apoptosis and migration capacity *in vitro*, but also enhance their survival and therapeutic potential for ischemic cardiovascular disease *in vivo*. (3) The beneficial effects generated from the hypoxia preconditioning of c-kit+ CPCs were mediated by the SDF-1α/CXCR4 axis signaling pathway and presented a SDF-1α/CXCR4 axis dependent manner.

In order to regenerate functional cardiac tissue, stem cell therapy has emerged as a promising strategy for resurrecting injured myocardium. Several stem cell populations such as embryonic stem cells, bone marrow mesenchymal stem cells (MSCs), endothelial progenitor cells, and skeletal myoblasts have been used to produce heart regeneration. We chose to use resident CPCs due to their incomparable cardiac regenerative capacity [27], [28]. Resident CPCs are multipotent, self-renewing, clonogenic and can directly differentiate into functional cardiomyocytes, endothelial cells and smooth muscle cells [7], [9], [10]. Moreover, these cells are endogenous normal components of the heart, they are responsible for the turnover of cardiomyocytes in the normal, aging and diseased heart. They can also rescue damaged myocardium by inducing cardiogenesis and neovascularization [3]–[6]. In addition, CPCs showed the ability for direct cardiomyocyte differentiation. Among different CPC populations, c-kit+ CPCs have presented better cardiac repair capability [4], [5], [10] and be considerated the most appropriated cell type for myocardial regeneration therapies [29]. c-kit is a tyrosine kinase receptor for stem cell factor (SCF) and the phosphorylation of c-kit is important for progenitor cell mobilization regulated by SDF-1/CXCR4 [30]. In addition, c-kit+ CSCs exert a paracrine survival effect on cardiomyocytes through induction of the IGF-1R and signalling pathway [29]. As shown in our study, an adequate amount of high purified c-kit positive CPCs could be obtained from an adult mouse heart via enzymatic digestion followed by magnetic-actived cell sorting.

Although c-kit+ CPCs appear to be the optimal choice for cell-based cardiac repair and regenerative therapy, they still pose a large challenge. Most of the transplanted cells die shortly after transplantation, therefore, developing a way for them to survive in the harsh environment after being engrafted into the infracted heart would be most beneficial [31]. Hypoxia preconditioning could stimulate serial adaptive cellular responses that would favor the survival of cells in infarct hearts after engraftment [32]. The primary target of hypoxia preconditioning is to activate the survival mechanisms inside the cell, and to better prepare the cells before transplantation. Different durations of hypoxia exposure would change a cell’s fate via cell signaling mechanisms. In order to determine the optimal duration for hypoxia preconditioning of mouse c-kit+ CPCs, we performed hypoxia time course experiments, resulting in 6 h being the optimal duration for hypoxia preconditioning of c-kit+ CPCs. As shown in the flow cytometry analysis, the apoptotic rate in the hypoxia 6 h group was 4.47%, the lowest among all groups. In addition, 6 h hypoxia preconditioned c-kit+ CPCs showed protective effects when these cells encountered prolonged severe hypoxia and serum deprivation. However, hypoxia treatment of c-kit+ CPCs for 24 h showed a significant increase in cell apoptosis (7.43%), which is similar with previous studies [33], [34]. Thus, hypoxia preconditioning of c-kit+ CPCs for 6 h was shown to be the optimal time course for our study.

The influences of hypoxia preconditioning on mouse c-kit+ CPCs were further examined by protein expression after hypoxia treatment for different time. The generation of pro-survival factors HIF-1α, Bcl-2, p-Akt, and VEGF significantly increased after hypoxia preconditioning for 6 h; possibly contributing to the enhanced cell anti-apoptosis and survival. In the present study, augmented anti-apoptosis and survival were demonstrated by the decreased annexin-V staining *in vitro* and reduced TUNEL labeling *in vivo*. The motogenic factor, SDF-1α, and its specific receptor, CXCR4, were also increased in c-kit+ CPCs after hypoxia treatment. Further verification of this phenomena was presented by the enhanced migration in the transwell system [10], [24].

Hypoxia preconditioning mimics the situation that the cells will meet when they are transplanted into the ischemic myocardium, and it better prepares cells for adapting the low oxygen tension environment in the ischemic heart after engraftment. Hu et al [17] reported that hypoxia preconditioning enhanced MSC survival and heart repair through up-regulated HIF-1α, growth factor and anti-apoptotic gene expression. In another study by Tang et al [10], hypoxia preconditioning of CPCs could increase CXCR4 expression and result in better homing and cardiac function after transplantation. Similar to the previously mentioned studies, our current study demonstrated that the expression of HIF-1α, CXCR4, anti-apoptotic gene Bcl-2, p-Akt, SDF-1α and VEGF were all elevated after hypoxia preconditioning, and was further verified by increased anti-apoptosis and migration capacity *in vitro* and decreased apoptosis and better cardiac rescue potency *in vivo*. These results indicated that SDF-1α and CXCR4 were activated in response to hypoxia stimuli, and could also activate the downstream Akt signaling pathway, which plays a key role in cell survival.

SDF-1α confers protection against myocardial ischemic injury [24], and plays a critical role in progenitor cell tissue retention, trafficking [35], and homing [10], [30]. It also has been shown to enhance the survival of progenitor cells in response to several stimuli such as ischemia/reperfusion injury, serum withdraw or apoptotic cell death [24], [36]. SDF-1α produces these protective effects through interaction with its specific receptor, CXCR4 [22]. As shown in this study, the expression of SDF-1α and CXCR4 in c-kit+ CPCs was elevated after hypoxia preconditioning. When exposed to severe hypoxia and serum deprivation, apoptosis decreased in hypoxia preconditioned c-kit+ CPCs compared to normoxia-cultured c-kit+CPCs. These protective effects were mostly abolished when hypoxia preconditioned CPCs were cultured with CXCR4 selective antagonist AMD 3100. Similar results were also evident *in viv*o, and the enhanced cardiac function improvement was blocked when CXCR4 specific antagonist AMD3100 was added during hypoxia preconditioning.

AMD3100 is a specific antagonist to the CXCR4 which is a membrane receptor, and AMD3100 binds competitively to CXCR4, effectively blocking>90% of SDF-1α binding [37]. Similarly, a recent study conducted by Stamatopoulos B et al. has presented a vigorous evidences that an AMD3100 concentration of 5 µg/ml can effectively binding to CXCR4, and suggested that 5 µg/ml AMD3100 can efficiently interfere with the SDF-1α/CXCR4 axis [38]. Collectively, these data indicate that the beneficial effects induced by hypoxia preconditioning of c-kit+ CPCs depend on the SDF-1α/CXCR4 axis.

Our study demonstrated that hypoxia preconditioning of c-kit+ CPCs led to increased cell survival and enhanced cardiac function improvement after MI. The beneficial effects induced by hypoxia preconditioning were mediated by the SDF-1α/CXCR4 axis. Hypoxia preconditioning may be a valuable strategy to augment the efficiency of cell-based cardiac regenerative therapy.

## Materials and Methods

### Ethics Statement

All animal studies were performed via a protocol approved by the Institutional Animal Care and Use Committee of Southeast University and complied with the National Research Council’s guidelines (approval ID: SYXK-2010.3908).

### Isolation and Culture of CPCs

CPCs were generated from the heart of two-month-old wild-type male C57BL/6 mice (Yangzhou Laboratory Animal Center). Hearts were harvested via a protocol approved by the Care of Experimental Animals Committee of Southeast University, Nanjing, China (Laboratory Animal Center of Southeast University). CPCs were isolated following the standard protocol as described previously, with minor modification [7]. After one to two weeks of growth, a layer of fibroblast-like cells was generated from the adherent cardiac explants over which small, round, phase-bright cells emerged. These phase-bright cells were collected using mild enzymatic digestion (0.05% trypsin at room temperature under direct visualization or no more than 3 minutes). Obtained cells were purified using magnetic-activated cell sorting with c-kit magnetic beads (Miltenyi Biotec Inc) as instructed by the protocols of the manufacturers. The obtained cells were seeded at 2×10^4^ cells/ml on poly-D-lysine (Sigma) coated dishes in cardiosphere growing medium (CGM; 35% IMDM/65%DMEM-Ham’s F-12 [Hyclone] mix containing 10% fetal calf serum [Hyclone], 2 mmol/L L-glutamine [Hyclone], 0.1 mmol/L 2-mecraptoethanol [Sigma], 2% B27 [Gibco], 5 ng/ml basic fibroblast growth factor (bFGF) [R&D], 10 ng/ml epidermal growth factor (EGF) [Peprotech], 40 nmol/L cardiotrophin-1 [Peprotech], 1 unit/ml thrombin [sigma], 100U/ml penicillinG [Hyclone], 100 µg/ml streptomycin [Hyclone]).

### Characterization of CPCs

c-kit+ CPCs were characterized using phase contrast microscopy evaluating morphology, immunocytochemistry for analyzing the expression of stem cell marker c-kit, and flow cytometry to examine the expression of stem cell surface markers. For immunostaining, cells were trypsinized and cultured on Matrigel coated dishes, fixed with 4% paraformaldehyde and stained with c-kit (1∶200) (Santa Cruz Biotech). In flow cytometry analysis, CPCs were trypsinized and re-suspended in phosphate buffered saline (PBS) and blocked with 3% FBS for 15 min. Cells were then labeled with PE-conjugated rat anti-mouse c-kit, FITC-conjugated rat anti-mouse Sca-1 (BD Biosciences) at 4°C in a dark room for 30 min. Cells were washed twice with cold PBS, and data was collected from 1×10^5^ cells on a FACSCalibur flow cytometer (BD Biosciences) and analyzed using WinMDI software.

### Hypoxic Preconditioning of CPCs

C-kit+ CPCs were divided into five groups: normoxia, hypoxia for 3 h, 6 h, 12 h and 24 h respectively. Hypoxia was achieved by placing the cells in a Modular Incubator Chamber (Billumps-Rothenberg; Del Mar, CA) according to the manufacturer’s instructions. After a brief time spent in the chamber, the cells were flushed with a mixture of 0.1%O_2_, 5%CO_2_ and 94.9%N_2_ for five minutes. The chamber was then closed and the cells incubated in 37°C for various lengths of time. Next, the cells were cultured under normoxic or hypoxic conditions for 3 h, 6 h, 12 h, and 24 h. The viability of c-kit+ CPCs was assessed by flow cytometry analysis using an Annexin V-FITC Apoptosis Detection Kit (Biouniquer). SDF-1α and vascular endothelial growth factor (VEGF) concentrations in the supernatant were determined by ELISA. After c-kit+ CPCs were cultured in hypoxic conditions for 6 h with or without CXCR4-selective antagonist AMD3100 (5 µg/ml)(Sigma), the anti-apoptosis, migration and cardiac repair potency were measured *in vitro* and *in vivo,* respectively.

### Effects of Hypoxic Preconditiong on CPCs Anti-apoptosis

The effect of hypoxic preconditioning on cell anti-apoptosis was assessed using flow cytometry analyses with an Annexin V-FITC Apoptosis Detection Kit (Biouniquer) according to the manufacturer’s instructions. Briefly, normoxia-cultured c-kit+ CPCs and hypoxic preconditioned c-kit+ CPCs with or without AMD3100 (5 µg/ml) were exposed to hypoxia for 48 h along with serum deprivation. Afterward, cell apoptosis was measured using a FACSCalibur (BD) and analyzed with CellQuest software (BD).

### CPCs Migration

A cell migration assay was performed in 24-well Transwell plates (8.0 µm, pore size) (Millipore, Billerica). The cells were seeded into the upper chamber of the Transwell system at a concentration of 2 ×10^4^cells/well in 100 µl medium, and the lower chamber was filled with 100 ng/ml SDF-1α (Sigma) in 600 µl medium. After 6 h of incubation at 37°C, 5% CO2, the upper sides of the filters were carefully washed with PBS, and cells remaining were removed with a cotton wool swab. The cells that migrated to the bottom side of the filter were fixed with 4% paraformaldehyde and stained using 0.1% crystal violet. The numbers of migrated cells were manually counted in three random fields per filter at ×200 magnification by a phase contrast microscope.

### Surgical Myocardial Infarction and CPCs Transplantation

Myocardial infarction (MI) was induced in adult C57BL/6 mice (weight 22–26 g, 10 weeks old) via permanent ligation of the left anterior descending (LAD) coronary artery as described previously [39]. Mice were anesthetized with an intraperitoneal injection of sodium pentobarbital (40 mg/kg) and mechanically ventilated (stroke volume 1.0 ml, ventilation rate 110/min). A left-side thoracotomy was performed in the fourth intercostal space, the LAD coronary artery was ligated 1.5 mm from the tip of the normal positioned left auricle with 8–0 silk suture, and infarction was visually verified by blanching in the anterior area of left ventricle just distal to the level of ligation. Mice were randomly distributed into the following four groups: control (n = 5), normoxia (n = 5), hypoxia (n = 5), and hypoxia + AMD3100 (n = 5). The later three groups received an intramyocardial injection of c-kit+ CPCs (5×10^5^ cells/mouse in 10 µl serum free DMEM), and were cultured under different conditions in the peri-infarct zone at two different sites using a sterile syringe with a 32G needle. The control group only received 10 µl serum free DMEM a few minutes after coronary artery ligation induced MI. The c-kit+ CPCs were labeled with GFP via incubation with AD-EGFP (MOI = 50, Invitrogen) for 16 h in complete medium before hypoxia preconditioning and the following transplantation.

### Echocardiography

Cardiac function was measured seven days post MI by transthoracic echocardiography (Vevo 770™; Visual Sonic, Toronto, Canada) and Vevo analysis software (Vevo 2.2.3; VisualSonics Inc). Mice were anesthetized by sodium pentobarbital (40 mg/kg, i.p.) with heart rate maintained at about 400 beats per minute. The hearts were imaged in Two-dimensional and M-mode echocardiography, and recordings were obtained from the left parasternal under the short-axis at the papillary muscles using a 35-MHz linear transducer. The Left ventricular end-diastolic volume (LVEDV), left ventricular end-diastolic diameter (LVEDD), left ventricular end-systolic volume (LVESV) and left ventricular end-systolic diameter (LVESD) were measured from at least three consecutive cardiac circles. Left ventricular ejection fraction (LVEF) and left ventricular fractional shortening (LVFS) were calculated via the following equations: LVEF =  (LVEDV - LVESV)/LVEDV × 100%, LVFS  =  (LVEDD - LVESD)/LVEDD × 100%.

### Histological Assessments

Mouse hearts were excised after flushing with PBS and the atrial tissue was removed. Body and heart weights were measured, then the cardiac tissues were fixed with 10% buffered formalin and embedded in paraffin. Four-micrometer sections were obtained for morphological analysis.

Masson’s trichrome staining was performed on the sections and assessed for infarct size; a calculated percentage of the left ventricular transverse circumference occupied by collagen. Cell apoptosis was detected by a terminal deoxynucleotidyl transferase–mediated dUTP-biotin nick end labeling (TUNEL) assay, and was performed using the In Situ Cell Death Detection Kit Fluorescein (Roche) according to the manufacturer’s instructions. TUNEL-positive nuclei were counted from five random fields near the area of infarction on each section.

### Western Blotting

Western blots were performed following standard protocols. An equal amount of cell lysates (40 µg protein) were denatured in 2 × SDS-PAGE sample buffer and electrophoresed for 3 h at 20 mA on 10% polyacrylamide gels. The separated proteins were transferred to a polyvinylidene difluoride (PVDF) membrane, and then the membranes were blocked with TBST solution (10 mM Tris-HCl, 150 mM NaCl, and 0.05% Tween 20) containing 5% nonfat dry milk for 4 h at room temperature. Next, they were incubated with the primary antibodies (1∶1000 dilution) and placed on a rocker at 4°C overnight. β-actin was used as the loading control (Santa Cruz, 1∶1000). The bound antibodies were detected with horseradish peroxide (HRP) conjugated sheep anti-rabbit IgG antibody on a rocker for 2 h at room temperature. Then the membranes were incubated with an enhanced chemiluminescence detection system for five minutes and imaged by a five minute film exposure. Protein expression was quantified by scanning densitometry.

### Statistical Analysis

Statistical analysis was performed via SPSS software (version 11.5; SPSS Inc.). All values are presented as mean ± SD. Differences between two groups were analyzed using the Student’s test, and differences between three or more groups were analyzed using one-way ANOVA and Bonferroni multiple comparison. All tests were two-tails and statistical significance was accepted if *P*<0.05.

## Supporting Information

Movie S1 Movie of representative differentiated CPCs with spontaneous beating derived from the cultured cardiac explants.
(AVI)
